# Biosynthesis of chiral 3-hydroxyvalerate from single propionate-unrelated carbon sources in metabolically engineered *E. coli*

**DOI:** 10.1186/1475-2859-9-96

**Published:** 2010-11-27

**Authors:** Hsien-Chung Tseng, Catey L Harwell, Collin H Martin, Kristala LJ Prather

**Affiliations:** 1 Department of Chemical Engineering, Massachusetts Institute of Technology, Cambridge, MA 02139, USA; 2 Synthetic Biology Engineering Research Center (SynBERC), Massachusetts Institute of Technology, Cambridge, MA 02139, USA; 3 Dow Chemical Company, Spring House, PA 19477, USA

## Abstract

**Background:**

The ability to synthesize chiral building block molecules with high optical purity is of considerable importance to the fine chemical and pharmaceutical industries. Production of one such compound, 3-hydroxyvalerate (3HV), has previously been studied with respect to the *in vivo *or *in vitro *enzymatic depolymerization of biologically-derived co-polymers of poly(3-hydroxybutyrate-co-3-hydroxyvalerate). However, production of this biopolymeric precursor typically necessitates the supplementation of a secondary carbon source (e.g., propionate) into the culture medium. In addition, previous approaches for producing 3HV have not focused on its enantiopure synthesis, and thus suffer from increased costs for product purification.

**Results:**

Here, we report the selective biosynthesis of each 3HV stereoisomer from a single, renewable carbon source using synthetic metabolic pathways in recombinant strains of *Escherichia coli*. The product chirality was controlled by utilizing two reductases of opposing stereoselectivity. Improvement of the biosynthetic pathway activity and host background was carried out to elevate both the 3HV titers and 3HV/3HB ratios. Overall, shake-flask titers as high as 0.31 g/L and 0.50 g/L of (*S*)-3HV and (*R*)-3HV, respectively, were achieved in glucose-fed cultures, whereas glycerol-fed cultures yielded up to 0.19 g/L and 0.96 g/L of (*S*)-3HV and (*R*)-3HV, respectively.

**Conclusions:**

Our work represents the first report of direct microbial production of enantiomerically pure 3HV from a single carbon source. Continued engineering of host strains and pathway enzymes will ultimately lead to more economical production of chiral 3HV.

## Background

The efficient production of enantiomerically pure chemicals from renewable resources has gained considerable attention especially in the fine chemical/pharmaceutical industry. Stereo-selective chemical processes generally employ expensive chiral catalysts, require harsh physical conditions and solvents, and suffer from extensive byproduct formation. In contrast, enzyme-catalyzed reactions are highly stereo-selective and can be performed in aqueous solutions under mild conditions [1]. As a result, replacing chemical processes by biological ones for the synthesis of chiral compounds has been extensively investigated not only due to superior stereo-selectivity of enzymatic reactions but also due to sustainability as an implementation of green chemistry [2-5]. One example is the production of hydroxyacids, a family of versatile chiral molecules containing one hydroxyl group and one carboxyl group [6]. These molecules have the potential to serve as useful chiral building blocks for a diverse range of products, including polyhydroxyalkanoates (PHAs) (biodegradable polymers) and optically-active fine chemicals, such as pharmaceuticals, vitamins, antibiotics, and flavor compounds [7-10]. Naturally, hydroxyacids are primarily found to be polymerized as PHAs where they serve as intracellular storage materials for numerous microbes. Those PHAs consist mostly of monomers with 3-hydroxy, 4-hydroxy, and 5-hydroxy groups with different lengths of main and side chains [11].

**Figure 1:**
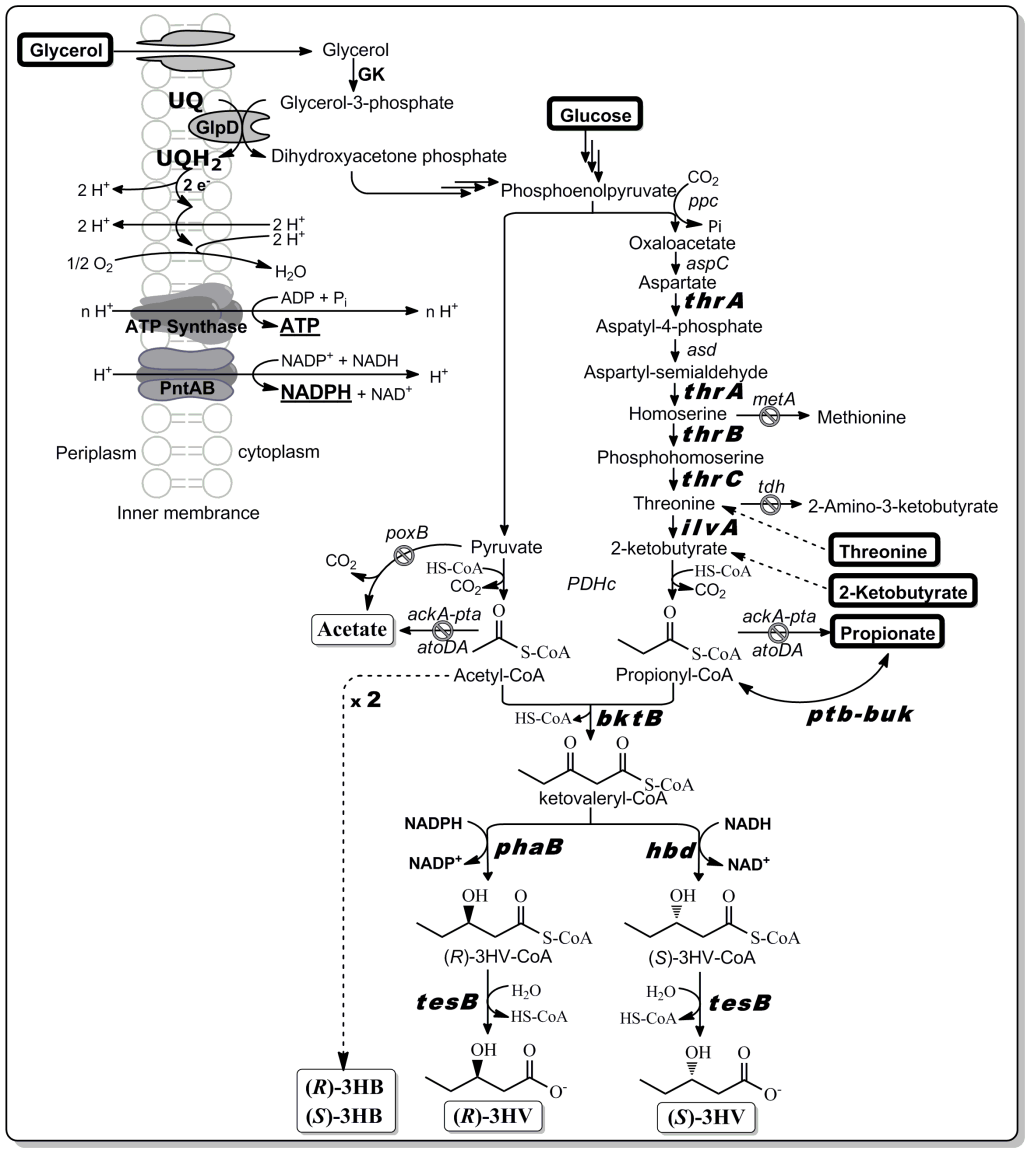
**Schematic representation of chiral 3HV production via the threonine biosynthesis pathway in metabolically engineered *E. coli***. Genes in bold are overexpressed while disrupted pathway steps are indicted by the "no" symbols. The carbon sources and main metabolic products in the system are enclosed by rectangular boxes with thick and thin lines, respectively. For glycerol utilization [43,44], a glycerol kinase (GK) phosphorylates glycerol to glycerol-3-phosphate, followed by oxidation to dihydroxyacetone phosphate that enters glycolysis. The oxidation reaction is catalyzed by a membrane enzyme called glycerol-3-phosphate dehydrogenase (GlpD) with concomitant production of ubiquinol (UQH_2_) from ubiquinone (UQ). Electrons stored in the ubiquinol are then transferred through the aerobic respiratory chain coupled with proton translocation from cytoplasm to periplasm. Both ATP and NADPH can be synthesized by an H^+^-driven proton movement from periplasm to cytoplasm, catalyzed by an ATP synthase and a membrane-bound transhydrogenase (PntAB), respectively.

Among the hydroxyacid monomers, 3-hydroxybutyrate (3HB) is the most prolific, with several reports on engineering *E. coli *for its production from renewable feedstocks [5,12-15]. Biosynthesis of 3HB begins with the condensation of two acetyl-CoA molecules, a commonly found cellular metabolite regardless of carbon source (Figure 1). However, economically-feasible production of longer-chain hydroxyacids is complicated by issues such as low yields and high prices of feedstocks due to the need to supplement a second carbon source. One example of such hydroxyacids is 3-hydroxyvalerate (3HV). The production of 3HV has been realized by the hydroxylation of valeric acid through fermentation of *Candida rugosa *[16]. It has also been reported that 3-hydroxyvaleronitrile can be converted into 3HV using the nitrilase activity of *Comamonas testosteroni *[17]. More recently, direct biological production of 3HV was demonstrated using recombinant *P. putida *KT2440 and levulinic acid as substrate, although the levulinic acid metabolism pathway in *P. putida *KT2440 has not yet been fully elucidated [18]. In the aforementioned cases, valeric acid, 3-hydroxyvaleronitrile, and levulinic acid were supplied as secondary carbon sources (in addition to glucose). Additionally, the chirality and/or enantiopurity of the 3HV produced in the above-mentioned studies is unclear as they did not report whether the synthesized 3HV was in the *R*, *S*, or racemic form. Alternatively, 3HV can be obtained through either the *in vivo *or *in vitro *enzymatic depolymerization of synthesized poly(3-hydroxybutyrate-co-3-hydroxyvalerate) (PHBV), a well known biodegradable polymer marketed as Biopol™ which is produced by the natural PHA accumulating bacterium *Ralstonia eutropha *when grown on glucose and propionate [19]. The production of PHBV has also been reported in recombinant *E. coli *upon introduction of the PHA biosynthesis genes of *R. eutropha *and when grown in glucose medium supplemented with valine or threonine [20]. Regardless of whether the end product is 3HV or PHBV, it can be generally concluded that supplementation of a second carbon source, such as valeric acid, 3-hydroxyvaleronitrile, levulinic acid, propionate, valine, or threonine in addition to glucose, is necessary to provide the 5-carbon unit precursor of 3HV. Unfortunately, the high price and/or toxicity of the added second carbon sources could limit industrial production of 3HV [21]. Therefore, synthesis of 3HV from a single carbon source has been proposed as an efficient and sustainable avenue in contrast to the above-mentioned systems.

A novel pathway for the production of PHBV solely from glycerol has been established in recombinant *Salmonella enterica *Serovar Typhimurium, containing a heterologous pathway that converts succinyl-CoA to propionyl-CoA, the essential precursor molecule of 3HV-CoA in PHBV synthesis [22]. However, expensive cyanocobalamin (CN-B_12_) was supplemented to the medium to provide the precursor of coenzyme B_12 _required for the activity of one of the enzymes in the B_12_-dependent biosynthetic pathway. It should also be noted that the pathway only functioned in *S. enterica*, a pathogen, but not *E. coli*, thus limiting its applicability to other industrially-relevant host organisms. In this study, we proposed an alternative biosynthetic pathway that does not require coenzyme B_12 _for its functionality to synthesize 3HV from glucose or glycerol. Specifically, we metabolically engineered *E. coli *to exploit its native metabolism for endogenous supply of propionyl-CoA via the threonine biosynthesis pathway, and introduced a heterologous pathway for chiral 3HV biosynthesis using acetyl-CoA and propionyl-CoA as precursor molecules. As stated above, several previous methods for producing 3HV did not focus on enantiopure synthesis. Similarly, due to the stereospecific constraints of PHBV synthesis, in which polymers are composed exclusively of (*R*)-3HB and (*R*)-3HV monomer units, the synthesis of (*S*)-3HV from PHBV remains effectively impossible. On the contrary, our proposed pathway makes possible the direct synthesis of both enantiomerically pure (*R*)-3HV and (*S*)-3HV.

We have identified a pathway which combines elements of our previously developed chiral 3HB biosynthesis pathway together with the natural threonine biosynthesis pathway of *E. coli *for direct biosynthesis of chiral 3HV (Figure 1). In the proposed pathway, chiral 3HV is produced from direct hydrolysis of 3HV-CoA catalyzed by a thioesterase II (encoded by *tesB*) where 3HV-CoA is obtained from condensation of one acetyl-CoA and one propionyl-CoA to form 3-ketovaleryl-CoA catalyzed by a thiolase (encoded by *bktB*), followed by a reduction of the 3-ketovaleryl-CoA to 3HV-CoA catalyzed by a 3-hydroxybutyryl-CoA dehydrogenase. Here, two enantio-selective 3-hydroxybutyryl-CoA dehydrogenases were utilized to control the chirality of 3HV-CoA produced. The NADPH-dependent dehydrogenase encoded by *phaB *produces (*R*)-3HV-CoA while the NADH-dependent dehydrogenase encoded by *hbd *produces (*S*)-3HV-CoA. It should be noted that in order to yield the highest 3HV titers and 3HV/3HB ratios, BktB was used as the thiolase in this study as opposed to other thiolases such as PhaA from *R. eutropha *H16 or Thil from *C. acetobutylicum *ATCC 824 because BktB has been shown to have highest *in vitro *enzyme activity towards the C_5 _substrate while PhaA and Thil were specific towards the C_4 _substrate [19]. Next, a pathway allowing for endogenous propionyl-CoA synthesis from glucose or glycerol, through the threonine metabolic pathway intermediate 2-ketobutyrate, was introduced to circumvent the need for feeding propionate. To examine the upstream pathway for endogenous supply of propionyl-CoA, we used a bottom-up approach where 2-ketobutyrate and threonine were, at first, fed to provide propionyl-CoA, in addition to glucose, to support 3HV production. In the final stage, a single carbon source of glucose or glycerol was used to provide both acetyl-CoA and propionyl-CoA to support 3HV biosynthesis in our metabolically engineered *E. coli*.

Overall, in this study we successfully demonstrated the direct biological production of enantiomerically pure (*R*)-3HV and (*S*)-3HV from a single carbon source. Improvements of the biosynthetic pathway and *E. coli *host strains have also been carried out to elevate 3HV titers and 3HV/3HB ratios.

## Results

### 3HV Synthesis from Glucose and propionate

Acetyl-CoA is an obligate central intermediate occurring in any organism and under any physiological condition; however, this is not the case for propionyl-CoA, which is only synthesized under special physiological conditions and from only few substrates [23]. Therefore, synthesis of 3HV-CoA requires propionyl-CoA biosynthesis. To validate our 3HV biosynthesis pathway, propionate was initially fed to provide propionyl-CoA as a precursor molecule to ensure the downstream pathway was capable of making chiral 3HV. It has been reported that the *R. eutropha *PHA biosynthesis genes can be functionally expressed in *E. coli*, resulting in homopolymer PHB production from glucose [24]. However, low levels of 3HV monomer within the synthesized co-polymer PHBV was observed in recombinant *E. coli *when propionate was co-fed with glucose in a way analogous to the procedure used for *R. eutropha *[24]. One explanation for the low content of 3HV monomer is that *E. coli *does not possess an efficient system for importing and/or converting propionate to propionyl-CoA. Therefore, to address the propionate utilization problem, a CoA-activation mechanism (encoded by the *ptb-buk *operon [25]) was incorporated into our previously developed 3HB pathway to investigate the substrate elasticity of the pathway for 3HV production.

**Figure 2:**
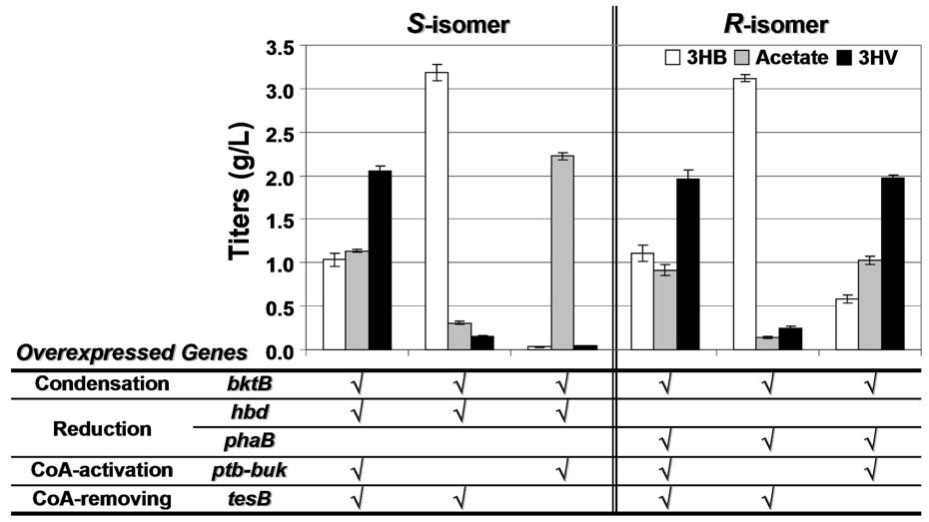
**3HV biosynthesis from glucose and propionate**. This figure shows shake-flask production of chiral 3HV by recombinant *E. coli *strain HCT 10 grown in LB supplemented with 20 g/L glucose and 20 mM sodium propionate. Over-expressed genes are indicated in the table below the graph.

Our results show that, in the absence of the CoA-activation mechanism, i.e. Ptb-Buk, only trace amount of 3HV was produced (Figure 2). On the contrary, introducing Ptb-Buk into the pathway yielded up to 2 g/L of both enantiomers of 3HV. It was noted that for strains expressing Ptb-Buk but leaving out TesB, only (*R*)-hydroxyacids (when PhaB was employed) were produced, consistent with a previous report that Ptb-Buk forms a reversible, stereo-seletive enzyme system [13]. Overall, these results indicate that CoA-activation was crucial for propionate utilization and, most importantly, all enzymes originally utilized for 3HB biosynthesis were able to catalyze synthesis of C_5 _molecules.

### 3HV Synthesis from Glucose and 2-Ketobutyrate

**Figure 3:**
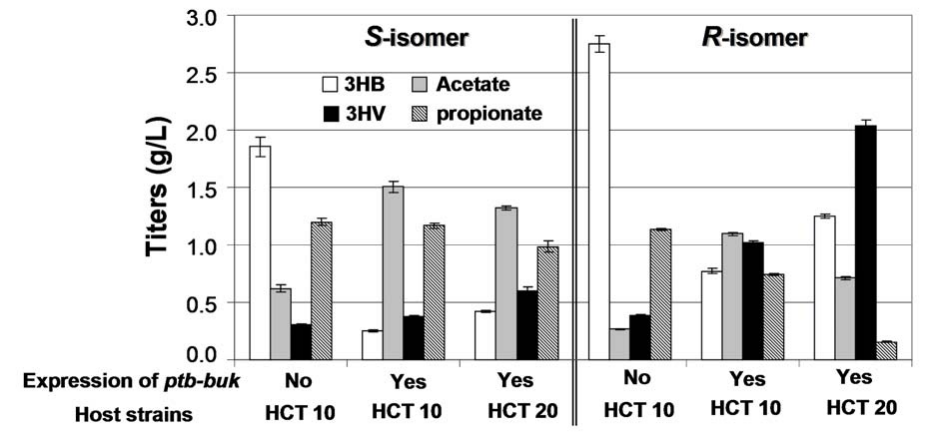
**3HV biosynthesis from glucose and 2-ketobutyrate**. This figure shows shake-flask production of chiral 3HV by recombinant *E. coli *grown in LB supplemented with 20 g/L glucose and 3 g/L sodium 2-ketobutyrate. Effects of overexpressing *ptb-buk *and using acetate pathway knockout strain HCT 20 (with additional deletions of *ackA-pta*. *poxB*, and *atoDA *genes compared to HCT 10) on 3HV production are compared. All strains contained the same set of plasmids pET-PB-B, and pCDF-T-H (for (*S*)-3HV synthesis) or pCDF-T-P (for (*R*)-3HV synthesis).

Propionyl-CoA can also be produced from 2-ketobutyrate, a common keto-acid intermediate for isoleucine biosynthesis, by the action of the endogenous pyruvate dehydrogenase complex enzyme (encoded by *PDHc*) (Figure 1) [26]. We first compared 3HV production from glucose and 2-ketobutyrate using pathways with and without over-expression of the *ptb-buk *operon. The results showed that the presence of Ptb-Buk reduced production of propionate (only observed in the *R*-isomer construct) and 3HB while increasing production of acetate and 3HV, yielding (*S*)-3HV and (*R*)-3HV with titers up to 0.38 g/L and 1.02 g/L, respectively (Figure 3). The increased production of acetate and 3HV was presumably due to the promiscuous activity of Ptb-Buk on cleaving excess acetyl-CoA and activating excess propionate. Given that 3HB production is a second-order reaction that should have a rate proportional to the square of the concentration of acetyl-CoA, a reduced acetyl-CoA pool resulting from the promiscuous activity of Ptb-Buk likely caused a significant decrease in 3HB production. In addition, propionyl-CoA is a competing substrate for BktB, so an increase in propionyl-CoA concentration may also reduce 3HB production.

In an effort to decrease acetate and increase 3HV production, several genes, including *atoDA *(encoding acetoacetyl-CoA transferase), *poxB *(encoding pyruvate oxidase), and *ackA-pta *(encoding acetate kinase and phosphate acetyltransferase) were deleted, and the resulting strain was designated as HCT 20. The production of (*S*)-3HV and (*R*)-3HV was further boosted to titers of 0.60 g/L and 2.04 g/L, respectively, in the recombinant acetate pathway knockout strains (HCT 20). In general, those strains produced less acetate and propionate and yielded more 3HB and 3HV compared to strains without these mutations (based on HCT 10), probably due to preserved acetyl-CoA and propionyl-CoA pools as a result of the introduced mutations. An empty-plasmid control experiment has also been conducted in the strain HCT 20 (that was not introduced with the 3HV pathway), yielding only trace amounts of acetate and propionate when grown in LB supplemented with glucose and 2-ketobutyrate (data not shown). This indicates that the production of acetate and propionate in the recombinant HCT 20 was attributed to the introduced CoA-cleaving activity conferred by *ptb-buk *and *tesB*.

### 3HV Synthesis from Glucose and Threonine

**Figure 4:**
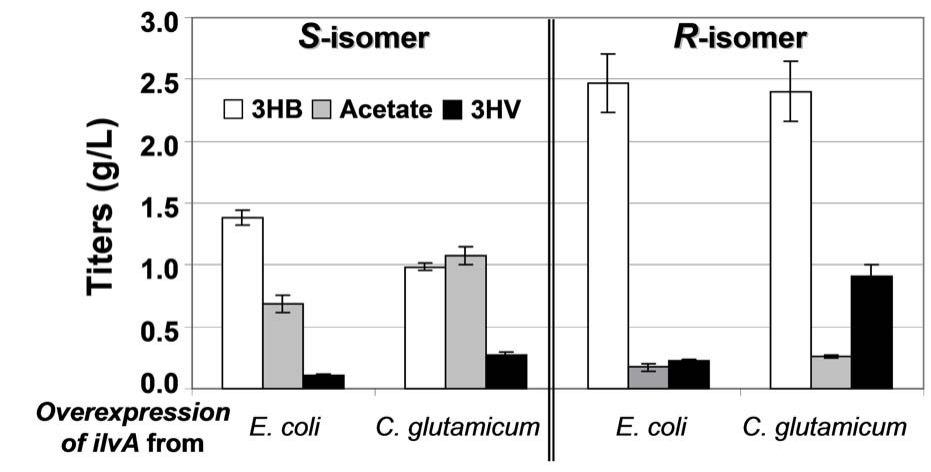
**3HV biosynthesis from glucose and threonine**. This figure shows shake-flask production of chiral 3HV by recombinant *E. coli *strain HCT 10 grown in LB supplemented with 20 g/L glucose and 3 g/L threonine. Chiral 3HV production using alternative threonine deaminases (encoded by *ilvA*) from *E. coli *and *C. glutamicum *is compared. All strains contained the same set of plasmids pET-PB-B, pCOLA-Icg or pCOLA-Iec as indicated, and pCDF-T-H (for (*S*)-3HV synthesis) or pCDF-T-P (for (*R*)-3HV synthesis).

The metabolic intermediate 2-ketobutyrate can be produced from threonine by the action of threonine deaminase. Co-feeding of threonine with glucose, together with over-expression of *E. coli *threonine deaminase (encoded by *ilvA*), was able to achieve production of (*S*)-3HV and (*R*)-3HV with titers up to 0.11 g/L and 0.22 g/L, respectively (Figure 4). Given that *E. coli *threonine deaminase is subject to feedback inhibition by isoleucine, a feedback resistant gene from *Corynebacterium glutamicum *[27] was also used, and the production of (*S*)-3HV and (*R*)-3HV was further boosted to titers of 0.27 g/L and 0.91 g/L, respectively, under the same culture conditions. This experiment has also been conducted in the recombinant acetate pathway knockout strains (HCT 20); however, no improvement in production of 3HB and 3HV was observed (data not shown).

### 3HV Synthesis from Glucose

We have demonstrated the production of chiral 3HV from glucose supplemented with propionate, 2-ketobutyrate, or threonine, in recombinant *E. coli*. The next step is to construct a threonine over-producing strain in an attempt to achieve 3HV biosynthesis from a single carbon source. To do so, we up-regulated the threonine biosynthesis pathway by over-expressing the *thrABC *opeon, cloned from the wild type *E. coli *or the threonine producer *E. coli *ATCC 21277 that has a single amino acid alteration in the homoserine dehydrogenase (encoded by *thrA*^G1297A^) for relieved feedback-inhibition [28]. Transcriptional attenuation of those genes was removed by replacing the native promoter with a T7*lac *promoter, allowing for IPTG-inducible expression. In addition, the pathways that compete with threonine formation as well as degrade threonine were eliminated by knocking out *metA *(encoding homoserine O-succinyltransferase) and *tdh *(encoding threonine dehydrogenase) genes, yielding strain HCT 21.

**Figure 5:**
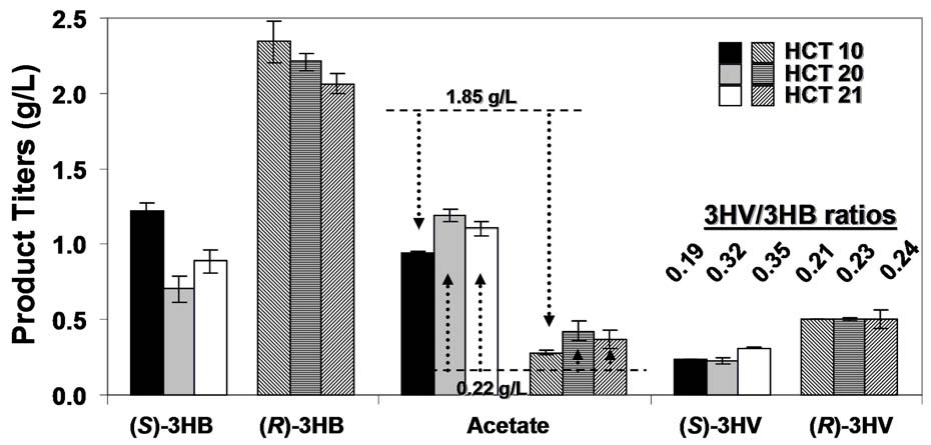
**3HV biosynthesis solely from glucose**. This figure shows **s**hake-flask production of chiral 3HV in various knock-out strains as described in Table 1. Cells were grown in LB supplemented with 20 g/L glucose. The top and bottom dashed lines represent the acetate titers produced from *E. coli *strain HCT 10 and HCT 20 harboring empty plasmids, respectively. All strains contained the same set of plasmids pET-PB-B, pCOLA-Tecm-Icg, and pCDF-T-H (for (*S*)-3HV synthesis) or pCDF-T-P (for (*R*)-3HV synthesis). The recombinant HCT 10 strains carrying an empty pCOLAduet-1 in place of pCOLA-Tecm-Icg, as control strains, produced essentially no 3HV (data not shown).

Our results showed that there was essentially no difference in 3HV production between strains expressing the wild type and feedback resistant *thrA *(data not shown) probably because threonine did not accumulate or its level was not high enough to exert a feedback inhibition to ThrA. We also compared 3HV production across three different *E. coli *strains, including HCT 10, HCT 20, and HCT 21. The mutants HCT 20 and HCT 21 carrying only empty plasmids significantly reduced acetate production to 0.22 g/L as opposed to 1.85 g/L by HCT 10 (Figure 5); however, recombinant mutant HCT 20 or HCT 21 containing the 3HV pathway produced as much acetate as the recombinant HCT 10, a counterintuitive finding (see Discussion). The deletions of *metA *and *tdh *enhanced (*S*)-3HV production by 41% (recombinant HCT 21 relative to recombinant HCT 20), but essentially had no effect on (*R*)-3HV production. Nevertheless, those mutations were able to boost the ratios of 3HV/3HB by decreasing the 3HB titers and/or increase the 3HV titers. Overall, titers as high as 0.31 g/L and 0.50 g/L of (*S*)-3HV and (*R*)-3HV were achieved in the recombinant HCT 21 with 3HV/3HB ratios up to 0.35 and 0.24, respectively (Figure 5).

### 3HV Synthesis from Glycerol

**Figure 6:**
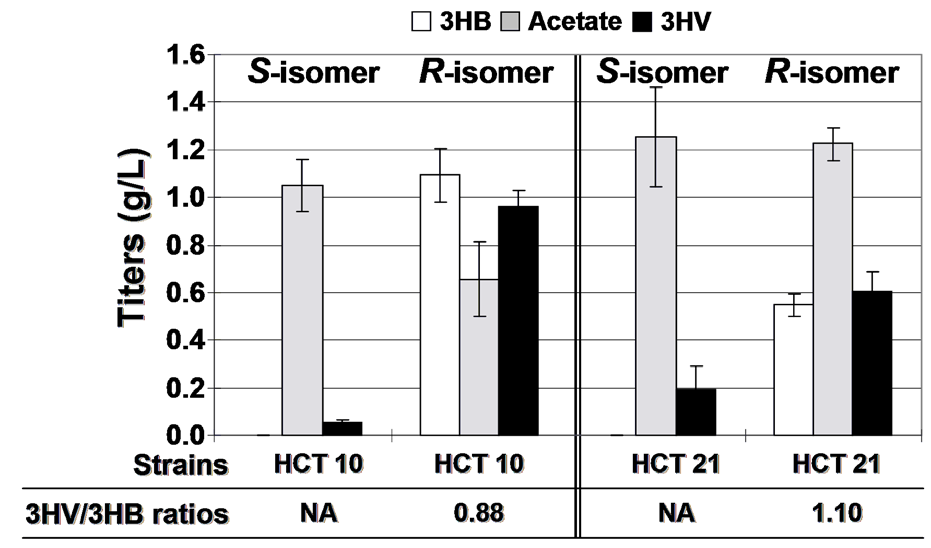
**3HV biosynthesis solely from glycerol**. This figure shows **s**hake-flask production of chiral 3HV in various knock-out strains as described in Table 1. Cells were grown in LB supplemented with 20 g/L glycerol. The amounts of (*S*)-3HB produced in both recombinant HCT 10 and HCT 21 strains were too low to be quantified due to a low detection limit by DAD at 210 nm; therefore, the 3HV/3HB ratios were not applicable (NA) to the (*S*)-isomer. All strains contained the same set of plasmids pET-PB-B, pCOLA-Tecm-Icg, and pCDF-T-H (for (*S*)-3HV synthesis) or pCDF-T-P (for (*R*)-3HV synthesis).

Glycerol has become a promising and abundant carbon source due to its generation as an inevitable byproduct of biodiesel production from vegetable oils or animal fats through a transesterification reaction [29]. There have been several reports on converting glycerol to more valuable compounds such as thymidine, ethanol, and 1,3-propanediol [30-32]. Glycerol is also more reduced than glucose, leading to a higher reduced cofactor pool in the cytoplasm [32]. Therefore, in addition to glucose, we investigated the ability of our recombinant *E. coli *to convert glycerol to chiral 3HV. Titers of 0.08 g/L and 0.96 g/L of (*S*)-3HV and (*R*)-3HV, respectively, were achieved in recombinant HCT 10, while recombinant HCT 21 produced 0.19 g/L and 0.60 g/L of (*S*)-3HV and (*R*)-3HV, respectively (Figure 6). As mentioned in the Materials and Methods section, in this specific experiment, concentration of 3HB was quantified by DAD at 210 nm that had a detection limit at around 0.08 g/L. As a result, the amounts of (*S*)-3HB produced in both recombinant HCT 10 and HCT 21 strains were too low to be quantified so that we could not report the 3HV/3HB ratios. Nonetheless, in the case of (*R*)-isomers, 3HV/3HB ratios could be as high as 0.88 and 1.10, respectively, in recombinant HCT 10 and HCT 21 strains. The high 3HV/3HB ratios can be beneficial in terms of product separation or biosynthesis of PHBV that enables high 3HV content.

### Confirmation of 3HV Stereochemistry

**Figure 7:**
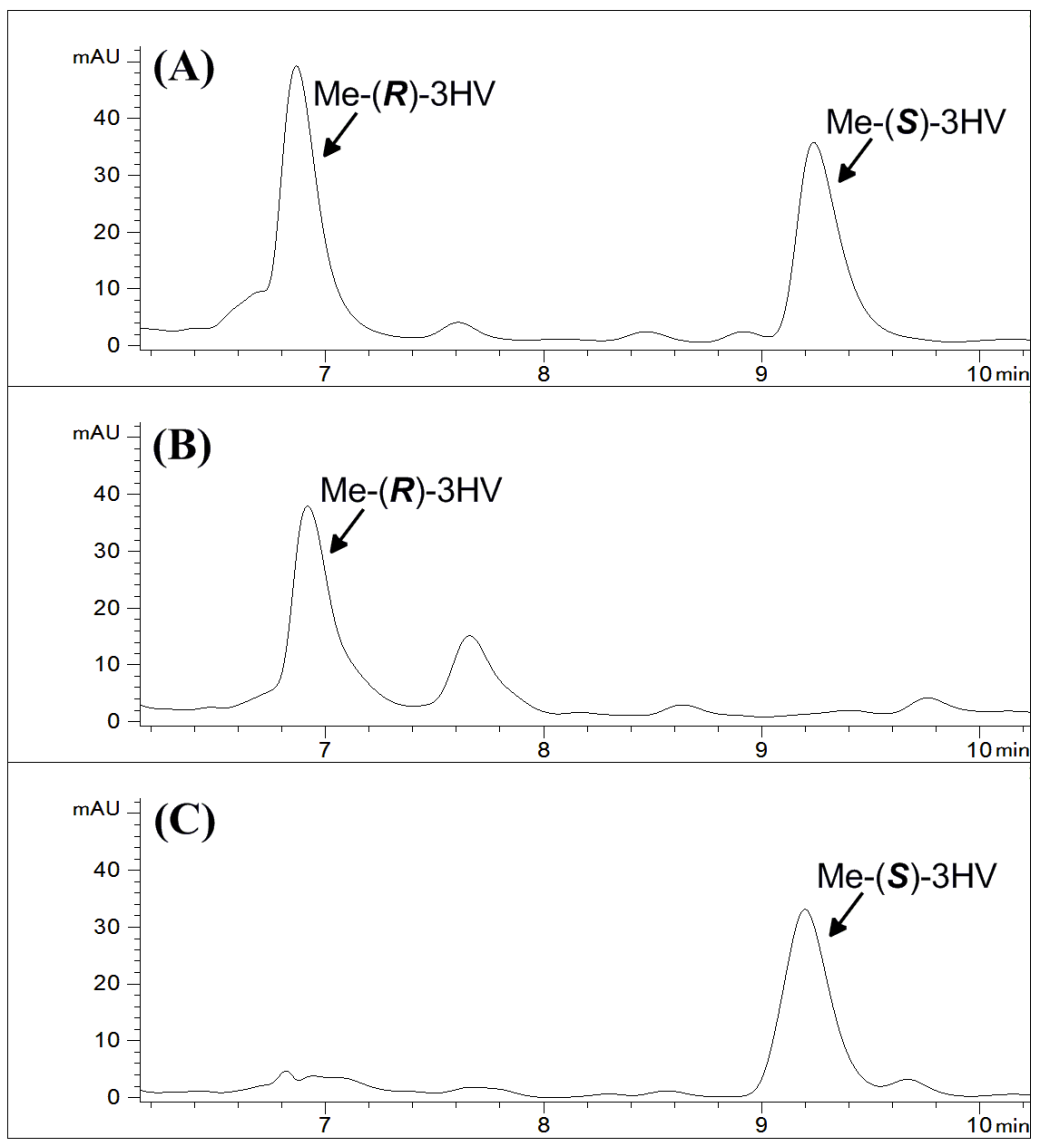
**Determination of the stereochemistry of 3HV**. HPLC spectra of (A) racemic 3HV standards after boiling in methanol, (B) culture medium from the recombinant strain HCT 10 expressing *bktB, phaB, tesB, and ptb-buk *after boiling in methanol, and (C) culture medium from the recombinant strain HCT 10 expressing *bktB, hbd, tesB, and ptb-buk *after boiling in methanol are shown.

The stereochemistry of the resulting 3HV in the media from these cultures was determined by methyl esterification of the 3HV present followed by chiral HPLC analysis using our previously developed method [15]. However, we could not assign an absolute stereochemistry to each sample due to the unavailability of enantiopure 3HV standards. However, based on our previous results regarding the product stereochemistry of *pha*B and *hbd *and the observation that Me-(*R*)-3HB has a faster retention time relative to Me-(*S*)-3HB, we expect Me-(*R*)-3HV to have a faster retention time than Me-(*S*)-3HV when analyzed by the same method. Thus, the 6.9 and 9.2 min peaks likely represent Me-(*R*)-3HV and Me-(*S*)-3HV, respectively (Figure 7). These results confirm the enantiopurity of biosynthesized 3HV.

## Discussion

In general, two approaches can be taken to engineer *E. coli *for direct 3HV production via the threonine biosynthesis pathway. The first is to utilize an existing threonine producer, such as *E. coli *ATCC 21277 [33], followed by further engineering to introduce our constructed 3HV pathway. However, this and other available threonine producing strains have typically been developed through multiple rounds of random mutagenesis and selection due to the difficulty of engineering this highly regulated and complex metabolic network. Although there are several successful cases in developing industrial threonine producers by such approaches, resultant strains usually also suffer from undesired phenotypes including, for example, growth retardation, low transformation efficiency, and by-product formation as a result of random mutations [34]. In addition, other uncharacterized mutations may hinder further strain development as often needed. Fortunately, recent advances in computational genomics have allowed for rational development of production strains [34]. Therefore, as a second approach, a genetically-defined threonine producing strain was established and introduced with the 3HV pathway to achieve direct microbial production of chiral 3HV from glucose or glycerol.

As seen in Figure 5 acetate is the major byproduct to the production of hydroxyacids (3HB and 3HV). In an effort to decrease acetate and increase 3HV production, a mutant strain HCT 20 with deletions on *atoDA*, *poxB*, and *ackA-pta *genes was developed. Counter-intuitively, the recombinant acetate pathway knockout strains of HCT 20 and HCT 21 produced slightly more acetate and less 3HB than recombinant HCT 10. We suspected that the enzymatic activity responsible for acetate production was restored by Ptb-Buk and TesB in the recombinant HCT 20 and HCT 21. In fact, in a separate experiment, both enzymes were found to have CoA-removing activities on acetyl-CoA and propionyl-CoA (data not shown), so an introduction of TesB and/or Ptb-Buk to strains HCT 20 or HCT 21 would likely restore the ability to produce acetate.

Apparently, knocking out enzymes responsible for acetate production failed to reduce acetate synthesis. Alternatively, to alleviate the substrate promiscuity of TesB and Ptb-Buk on acetyl-CoA, and thus reduce acetate production, one approach called enzyme co-localization could be implemented to allow substrate channeling between enzymes [35]. For example, pathway enzymes of Hbd and TesB, catalyzing successive reactions, can be co-localized in an attempt to reduce the amount of freely floating TesB that may hydrolyze acetyl-CoA as well as to increase accessibility of 3HV-CoA by TesB. The spatial organization of the enzymes can be achieved using either the leucine zipper, a dimer resulting from interaction between leucine residues [36], or the synthetic scaffolds, constructed from protein-protein interaction domains [37]. Furthermore, expressing enzymes that would assimilate produced acetate is another way to reduce acetate accumulation. For example, acetyl-CoA synthetase (encoded by *acs*) from *E. coli *can be over-expressed to convert acetate to acetyl-CoA with the use of one ATP. While successfully demonstrated in one work [38], in our case, over-expression of *acs *was found to have essentially no effect on acetate reduction (data not shown). Additionally, to overcome the hurdle of acetate reduction, approaches like protein engineering of TesB and/or Ptb-Buk to alleviate their substrate promiscuity, or utilization of better isozymes with more stringent substrate specificity could also mitigate the carbon loss in the form of acetate.

Among microbes, NADH and NADPH play a central role in energy metabolism by providing the cell with the reducing power for a variety of cellular redox reactions. The availability of such cofactors could impose a huge impact on the functionality of introduced biosynthetic pathways. In fact, we have previously shown that the NADPH/NADP^+ ^ratio was two- to three-fold higher than the NADH/NAD^+ ^ratio under the culture conditions examined, presumably affecting *in vivo *activities of PhaB and Hbd and resulting in greater production of (*R*)-3HB than (*S*)-3HB [15]. Given that our proposed 3HV pathway was based on the previously established 3HB pathway, it was also expected to see the same trend of greater production of (*R*)-3HV than (*S*)-3HV, even though the cofactor dependency of 3HV synthesis may be complicated by the energetically expensive threonine biosynthesis pathway with utilization of both ATP and NADPH. In an effort to perturb the cofactor balance within the cells, thereby tuning the production of (*R*)-3HV and (*S*)-3HV, we attempted to used glycerol, a promising, abundant, and highly-reduced carbon source, to support 3HV production. Based on our calculation of reducing equivalents (e^-^) of glucose and glycerol, on the same basis of 2 moles of phosphoenolpyruvate synthesized, glucose and glycerol possess, respectively, 24 and 28 reducing equivalents. Potentially, the additional four reducing equivalents can be utilized to generate two NADPH or equivalent amount of ATP. In fact, it has been experimentally confirmed that a higher intracellular NADPH/NADP^+ ^ratio was observed when glycerol was used as a carbon source than glucose, and this higher ratio was also reflected in boosted production of thymidine as its biosynthesis requires NADPH as a cofactor [32]. Given that both NADPH and ATP play a central role in threonine biosynthesis, we hypothesized that the use of glycerol, which could generate more NADPH and ATP (Figure 1) relative to glucose, may favor threonine biosynthesis by directing more carbon flux towards production of propionyl-CoA, thus favoring the formation of 3HV relative to 3HB. In agreement with our hypothesis, a higher 3HV/3HB ratio was obtained in the (*R*)-3HV production when glycerol was used as the carbon source (Figure 6). In addition, much larger ratios of the total (R)-hydroxyacids (summation of (R)-3HB and (R)-3HV titers) to the total (S)-hydroxyacids (summation of (S)-3HB and (S)-3HV titers) were observed in glycerol-fed cultures (Figure 6) compared to glucose-fed cultures (Figure 5). We hypothesize that the higher intracellular NADPH/NADP+ ratio as a result of the use of glycerol would favor (R)-hydroxyacid biosynthesis compared to the use of glucose, thus yielding the larger ratios of total (R)-hydroxyacids to total (S)-hydroxyacids.

As mentioned previously, BktB was chosen as the primary thiolase due to its high enzymatic specificity towards the C_5 _substrate. Given that *E. coli *has an endogenous thiolase (encoded by *atoB*), a deletion of *atoB *was expected to increase the ratio of 3HV/3HB as AtoB has been shown to prefer to condense two molecules of acetyl-CoA instead of one propionyl-CoA and one acetyl-CoA [19]. However, our preliminary result showed that the recombinant HCT 11 with an *atoB *deletion behaved exactly as the recombinant HCT 10, and the deletion in *atoB *had essentially no effect on 3HV production (data not shown), implying that *atoB *may not be a constitutively expressed gene. In addition, it is noteworthy that increased 3HV production in the recombinant HCT 20 relative to the recombinant HCT 10 was observed only with 2-ketobutyrate supplementation (Figure 3) but not with the threonine supplementation, solely glucose, or solely glycerol experiments (Figure 4, 5 and 6); similarly, an accumulation of propionate only occurred in the 2-ketobutyrate supplementation experiment (Figure 3), altogether, indicating that 3HV biosynthesis from glucose or glycerol is most likely limited by the precursor propionyl-CoA. Therefore, approaches to increase the availability of propionyl-CoA could enhance the 3HV production.

## Conclusions

Carbon skeletons with even-chain number are naturally found in fatty acid metabolism, but those with odd-chain number are pretty novel. As a result, there is a good deal of interest in making odd-carbon chain molecules such as 3HV (C5) and propionate (C3) because they are so much harder to get to than even-carbon chain ones such as acetate (C2) and butyrate/butanol (C4). This paper opens the way for biosynthesis of the odd-carbon chain molecules from renewable feedstocks. Taking together, our work represents the first report of direct microbial production of enantiomerically pure 3HV from a single carbon source. In addition, we have explored the production of each stereoisomer of 3HV across different genetically altered *E. coli *strains, along with various enzyme homologs, for enhanced chiral 3HV production. Further engineering of host strains and pathway enzymes should lead to higher 3HV titers and a more economical bioprocess for the production of chiral 3HV.

## Methods

### Microorganisms

The bacterial strains used are listed in Table 1. *C. acetobutylicum *ATCC 824, *C. glutamicum *ATCC 13032, and a threonine hyper-producer *E. coli *ATCC 21277 were purchased from the American Type Culture Collection (ATCC, Manassas, VA). *R. eutropha *H16 was provided by Professor Anthony Sinskey of the Department of Biology at the Massachusetts Institute of Technology (Cambridge, MA, USA). *E. coli *DH10B (Invitrogen, Carlsbad, CA) and ElectroTen-Blue (Stratagene, La Jolla, CA) were used for transformation of cloning reactions and propagation of all plasmids. MG1655 (kindly donated by Professor Gregory Stephanopoulos of the Department of Chemical Engineering at the Massachusetts Institute of Technology, USA) was used as the parental strain for genetic modification. Host gene deletions of *endA*, *recA*, *atoDA*, *ackA-pta*, *poxB*, *tdh*, *metA*, and *atoB *were achieved with P1 transduction using the Keio collection strains as donor cells [39]. The kanamycin cassette was removed using plasmid pCP20 as described by Datsenko and Wanner [40] and the successfully constructed mutant strains were verified by colony PCR using appropriate primers. Strains carrying a λDE3 lysogen were constructed using a λDE3 Lysogenization Kit (Novagen, Darmstadt, Germany) for site-specific integration of λDE3 prophage into each host.

**Table 1:** *E. coli *strains, plasmids and oligonucleotides used

Name	Relevant Genotype	Reference
**Strains**		
DH10B	F^- ^*mcr*A Δ(*mrr-hsd*RMS-*mcr*BC) ϕ80*lac*ZΔM15 Δ*lacX74 recA1 endA1 araD139*Δ(*ara, leu*)7697 *galU galK *λ^- ^*rpsL nupG*	Invitrogen
ElectroTen-Blue	Δ(*mcrA*)*183 *Δ*(mcrCB-hsdSMR-mrr)173 endA1 supE44 thi-1 recA1 gyrA96 relA1 lac *Kan^r ^[F' *proAB lacI*^q^*Z*ΔM15 Tn*10 *(Tet^r^)]	Stratagene
MG1655	F^- ^λ^- ^*ilvG- rfb-50 rph-1*	ATCC 700926
HCT 10	MG1655 Δ*endA *Δ*recA *(DE3)	This study
HCT 11	MG1655 Δ*endA *Δ*recA *Δ*atoB *(DE3)	This study
HCT 20	MG1655 Δ*endA *Δ*ackA-pta *Δ*atoDA *Δ*poxB *(DE3)	This study
HCT 21	MG1655 Δ*endA *Δ*ackA-pta *Δ*atoDA *Δ*poxB *Δ*metA *Δ*tdh *(DE3)	This study
		
**Plasmids**		
pETDuet-1	ColE1(pBR322) *ori*, *lacI*, T7*lac*, Amp^R^	Novagen
pCDFDuet-1	CloDF13 *ori*, *lacI*, T7*lac*, Strep^R^	Novagen
pCOLADuet-1	COLA *ori*, *lacI*, T7*lac*, Kan^R^	Novagen
pET-B	pETDuet-1 harboring *bktB *from *R. eutropha *H16	This study
pET-PB-B ^a^	pETDuet-1 harboring *ptb*-*buk *operon from *C*. *acetobutylicum *ATCC 824, and *bktB *from *R. eutropha *H16	This study
pCDF-H	pCDFDuet-1 harboring *hbd *from *C*. *acetobutylicum *ATCC 824	This study
pCDF-T-H ^a^	pCDFDuet-1 harboring *tesB *from *E. coli *MG1655, and *hbd *from *C*. *acetobutylicum *ATCC 824	This study
pCDF-P	pCDFDuet-1 harboring *phaB *from *R. eutropha *H16	This study
pCDF-T-P ^a^	pCDFDuet-1 harboring *tesB *from *E. coli *MG1655, and *phaB *from *R. eutropha *H16	This study
pCOLA-Iec	pCOLADuet-1 harboring *ilvA *from *E. coli *MG1655	This study
pCOLA-Icg	pCOLADuet-1 harboring *ilvA *from *C. glutamicum*	This study
pCOLA-Tec-Icg ^a^	pCOLADuet-1 harboring *thrABC *operon from *E. coli *MG1655, and *ilvA *from *C. glutamicum *ATCC 13032	This study
pCOLA-Tecm-Icg ^a^	pCOLADuet-1 harboring *thrA*^G1297A^*BC *operon from *E. coli *ATCC 21277, and *ilvA *from *C. glutamicum *ATCC 13032	This study
		
**Primers**^**b**^	**Sequence 5'→3'**^**c**^	
bktB_US_EcoRI	*GAATTC*ATGACGCGTGAAGTGGTAGTG	Sigma-Genosys
bktB_DS_XhoI	*CTCGAG*CGCAAGGCTAACCTCAGAT	Sigma-Genosys
hbd_US_NdeI	ATT*CATATG*AAAAAGGTATGTGTTATAGG	Sigma-Genosys
hbd_DS_AvrII	ATT*CCTAGG*CAGGTCGACTCTAGAACTTA	Sigma-Genosys
phaB_US_MfeI	ATT*CAATTG*ACGAAGCCAATCAAGGAG	Sigma-Genosys
phaB_DS_AvrII	ATT*CCTAGG*GGTCAGCCCATATGCAG	Sigma-Genosys
tesB_US_NcoI	ATT*CCATGG*GCATGAGTCAGGCGCTAA	Sigma-Genosys
tesB_DS_NotI	ATT*GCGGCCGCG*ACTCTAGAGACTTAATTGTG	Sigma-Genosys
ilvAec_US_NdeI	ATTA*CATATG*GCTGACTCGCAAC	Sigma-Genosys
ilvAec_DS_AvrII	ATTA*CCTAGG*CATTTTTCCCTAACC	Sigma-Genosys
ilvAcg_US_NdeI	ATTA*CATATG*AGTGAAACATACGTGTC	Sigma-Genosys
ilvAcg_DS_AvrII	ATTA*CCTAGG*CCTTCAGCTATGTTTA	Sigma-Genosys
thrABC_US_BamHI	ATTA*GGATCC*AAGGAGATATATCATGCGAGTGTTGAAG	Sigma-Genosys
thrABC_US_NcoI	ATTA*CCATGG*GCATGCGAGTGTTGAAG	Sigma-Genosys
thrABC_DS_SalI	ATTA*GTCGAC*GATAATGAATAGATTTTACTGATG	Sigma-Genosys

^a ^Each gene is under the control of the T7*lac *promoter with a ribosome binding site.

^b ^Primers were synthesized at Sigma-Genosys, St. Louis, MO.

^c ^Restriction enzyme sites used in the cloning are shown in underlined italics.

### Plasmid Construction

Genes derived from *C. acetobutylicum *ATCC 824 (*hbd *and *ptb-buk *operon), *R. eutropha *H16 (*bktB *and *phaB*), *C. glutamicum *ATCC 13032 (*ilvA*), *E. coli *MG1655 (*tesB, ilvA, and thrABC *opeon), and *E. coli *ATCC 21277 (*thrA*^G1297A^*BC *opeon) were obtained by polymerase chain reaction (PCR) using genomic DNA (gDNA) templates. All gDNAs were prepared using the Wizard Genomic DNA Purification Kit (Promega, Madison, WI). Custom oligonucleotides (primers) were purchased for all PCR amplifications (Sigma-Genosys, St. Louis, MO) as listed in Table 1. In all cases, Phusion High Fidelity DNA polymerase (Finnzymes, Espoo, Finland) was used for DNA amplification. Restriction enzymes and T4 DNA ligase were purchased from New England Biolabs (Ipswich, MA). Recombinant DNA techniques were performed according to standard procedures [41]. Three co-replicable vectors, pETDuet-1, pCDFDuet-1, and pCOLADuet-1 (Novagen, Darmstadt, Germany), were used for construction of chiral 3HV biosynthetic pathways [42]. All vectors contain two multiple cloning sites (MCS), each of which is preceded by a T7*lac *promoter and a ribosome binding site (RBS), affording high-level expression of each individual gene.

Plasmids constructed in the present work are listed in Table 1. For cloning genes, PCR products incorporated with desired restriction sites within the 5' and 3' primers were digested, and the resulting DNA fragments were then cloned into pETDuet-1, pCDFDuet-1, or pCOLADuet-1. The *bktB *gene was inserted in between the *Mfe*I and *Xho*I sites (MCS II) of pETDuet-1 to create pET-B. The *ptb-buk *gene, digested from pCDF-PB with EcoRI and NotI [15], was inserted between the *EcoR*I and *Not*I sites (MCS I) of pET-B to create pET-PB-B. Plasmid pCDF-H was created by inserting the *hbd *gene between the *Nde*I and *Avr*II sites (MCS II) of pCDFDuet-1. Cloning the *tesB *gene between the *Nco*I and *Not*I sites (MCS I) of pCDFDuet-1 resulted in plasmid pCDF-T. Plasmid pCDF-T-H was then created by inserting the *hbd *gene between the *Nde*I and *Avr*II sites (MCS II) of pCDF-T. In a similar manner, plasmid pCDF-P was created by inserting the *phaB *gene between the *Mfe*I and *Avr*II sites (MCS II) of pCDFDuet-1. Plasmid pCDF-T-P was created by inserting the *phaB *gene between the *Mfe*I and *Avr*II sites (MCS II) of pCDF-T. Plasmids of pCOLA-Iec and pCOLA-Icg were constructed by inserting the *E. coli ilvA *and *C. glutamicum ilvA*, respectively, between the *Nde*I and *Avr*II sites (MCS II) of pCOLADuet-1. The *thrABC *operon from MG1655 was inserted in between the *Nco*I and *Sal*I sites (MCS I) of pCOLADuet-1 to create pCOLA-Tec. Plasmid pCOLA-Tec-Icg was then created by inserting the *C. glutamicum ilvA *gene between the *Nde*I and *Avr*II sites (MCS II) of pCOLA-Tec. To construct plasmid pCOLA-Tecm-Icg, the *thrA*^G1997A^*BC *operon from *E. coli *ATCC 21277 was inserted in between the *BamH*I and *Sal*I sites (MCS I) of pCOLA-Icg. All constructs were confirmed to be correct by restriction enzyme digestion and nucleotide sequencing.

### Culture Conditions

Seed cultures of the recombinant strains were grown in LB medium at 30°C overnight on a rotary shaker at 250 rpm. For the biosynthesis of chiral 3HV, the seed cultures were used to inoculate 50 mL LB medium supplemented with 20 g/L glucose or 20 g/L glycerol at an inoculation volume of 2% in 250 mL flasks. Cultures were then incubated at 30°C on a rotary shaker until OD_600 _reached 0.8~1.0. At this point, 1 mM IPTG was added to the cultures to induce recombinant protein expression. Following induction, cells were cultivated at 30°C and sampled at 24 h intervals for up to 72 h post-induction for HPLC analysis. We found that both 3HB and 3HV titers did not reach a plateau until 48 h and that there was essentially no difference in the titers between 48 h and 72 h. Accordingly, only the peak titers observed at 48 h were reported in this study. In some experiments as indicated, 20 mM (~1.92 g/L) sodium propionate, 3 g/L sodium 2-ketobutyrate, or 3 g/L threonine was added into the cultures at the same time of induction. In all cases, LB medium was supplemented with 50 mg/L ampicillin, 50 mg/L streptomycin, and 30 mg/L kanamycin, as appropriate. In general, experiments were performed in triplicates, and data are presented as the averages and standard deviations of the results.

### Metabolite Analysis

Samples were centrifuged to pellet cells while the aqueous supernatant was collected for HPLC analysis. Products of interest, including 3HB, 3HV, glucose, glycerol, 2-ketobutyrate, acetate, and propionate, were analyzed via HPLC using an Agilent 1100 series instrument equipped with a refractive index detector (RID) and a diode array detector (DAD). Given that the 3HB peak is overlapped with the glycerol peak in the RID chromatogram, detection of 3HB in the glycerol-fed cultures was achieved using the DAD at 210 nm. Analyte separation was achieved using an Aminex^® ^HPX-87 H anion exchange column (Bio-Rad Laboratories, Hercules, CA) with 5 mM H_2_SO_4 _as the mobile phase. The mobile phase was pumped at a constant rate of 0.6 mL/min, and the column and detector temperatures were each set at 35°C throughout. Concentrations were determined by linear extrapolation from calibration of external standards.

### Chiral Analysis of 3HV

The stereochemistry of 3HV produced was determined by methyl esterification of the 3HV present in the medium followed by chiral HPLC analysis as described in a previously reported method [15]. The chiral analysis was performed on an Agilent 1100 Series instrument equipped with a Chiralcel OD-H column (0.46 cm ϕ × 25 cm) purchased from Daicel Chemical Industries (West Chester, PA). Methyl-3HV was detected on a DAD at 210 nm. The mobile phase was 9:1 *n*-hexane:isopropanol and the flow rate through the column was 0.7 mL/min. Due to unavailability of standards of Methyl-(*R*)-3HV and Methyl-(*S*)-3HV, these spectra were compared to a racemic 3HV standard (Epsilon Chimie, Brest, France) derivatized by methyl esterification.

## Competing interests

The authors declare that they have no competing interests.

## Authors' contributions

HCT and KLJP initiated and coordinated the project. HCT performed experiments. CLH assisted HCT with gene cloning, cell culture, and data analysis. CHM performed the chiral analysis. HCT wrote and KLJP edited the paper. All authors approved the final version of the manuscript.

## References

[B1] Patel RN Stereoselective Biocatalysis 2000 Boca Raton, FL.: CRC Press

[B2] Tokiwa Y Calabia BP Biological production of functional chemicals from renewable resources Can J Chem 2008 86 548 555 10.1139/V08-046

[B3] Shiraki M Endo T Saito T Fermentative production of (*R*)-(-)-3-hydroxybutyrate using 3-hydroxybutyrate dehydrogenase null mutant of *Ralstonia eutropha *and recombinant *Escherichia coli* J Biosci Bioeng 2006 102 529 534 10.1263/jbb.102.529 17270718

[B4] Chen GQ Wu Q Microbial production and applications of chiral hydroxyalkanoates Appl Microbiol Biotechnol 2005 67 592 599 10.1007/s00253-005-1917-2 15700123

[B5] Zhao K Tian G Zheng Z Chen JC Chen GQ Production of *D*-(-)-3-hydroxyalkanoic acid by recombinant *Escherichia coli* FEMS Microbiol Lett 2003 218 59 64 12583898 10.1111/j.1574-6968.2003.tb11498.x

[B6] Ren Q Ruth K Th?ny-Meyer L Zinn M Enatiomerically pure hydroxycarboxylic acids: current approaches and future perspectives Appl Microbiol Biotechnol 2010 87 1 41 52 10.1007/s00253-010-2530-6 20393709 PMC2872024

[B7] Chiba T Nakai T A new synthetic approach to the carbapenem antibiotic PS-5 from ethyl(*S*)-3-hydroxybutanoate Chem Lett 1987 11 2187 2188 10.1246/cl.1987.2187

[B8] Seebach D Chow HF Jackson RFW Sutter MA Thaisrivongs S Zimmermann J (+)-11,11'-di-O-methylelaiophylidene-preparation from Elaiophylin and total synthesis from (*R*)-3-hydroxybutyrate and (*S*)-malate Liebigs Ann Chem 1986 1986 1281 1308 10.1002/jlac.198619860714

[B9] Chiba T Nakai TA Synthetic approach to (1)-thienamycin from methyl (*R*)-(2)-3-hydroxybutanoate. A new entry to (3*R*,4*R*)-3-[(*R*)-1-hydroxyethyl]-4-acetoxy-2-azetidinone Chem Lett 1985 161 651 654 10.1246/cl.1985.651

[B10] Mori K A simple synthesis of (*S*)-(+)-sulcatol, the pheromone of *Gnathotrichus tetusus *employing baker's yeast for asymmetric reduction Tetrahedron 1981 37 1341 1342 10.1016/S0040-4020(01)92449-4

[B11] Steinbuchel A Valentin HE Diversity of bacterial polyhydroxyalkanoic acids FEMS Microbiology Letters 1995 128 219 228 10.1016/0378-1097(95)00125-O

[B12] Liu Q Ouyang SP Chung A Wu Q Chen GQ Microbial production of *R*-3-hydroxybutyric acid by recombinant *E. coli *harboring genes of *phbA*, *phbB*, and *tesB* Appl Microbiol Biotechnol 2007 76 811 818 10.1007/s00253-007-1063-0 17609944

[B13] Lee SH Park SJ Lee SY Hong SH Biosynthesis of enantiopure (*S*)-3-hydroxybutyric acid in metabolically engineered *Escherichia coli* Appl Microbiol Biotechnol 2008 79 633 641 10.1007/s00253-008-1473-7 18461320

[B14] Lee SY Lee Y Metabolic engineering of *Escherichia coli *for production of enantiomerically pure (*R*)-(-)-hydroxycarboxylic acids Appl Environ Microbiol 2003 69 3421 3426 10.1128/AEM.69.6.3421-3426.2003 12788745 PMC161469

[B15] Tseng HC Martin CH Nielsen DR Prather KL Metabolic engineering of Escherichia coli for enhanced production of (R)- and (S)-3-hydroxybutyrate Appl Environ Microbiol 2009 75 3137 3145 10.1128/AEM.02667-08 19304817 PMC2681625

[B16] Hasegawa J HS Ogura M Watanabe K Production of beta-hydroxycarboxylic acids from aliphatic carboxylic acids by microorganisms J Ferment Technol 1981 59 257 262

[B17] Bramucci MG Dicosimo Robert Fallon Robert Gavagan John E Herkes Frank Wilczek Lech 3-Hydroxycarboxylic acid production and use in branched polymers United States Patent 7138480 2006

[B18] Martin CH Prather KLJ High-titer production of monomeric hydroxyvalerates from levulinic acid in Pseudomonas putida Journal of Biotechnology 2009 139 61 67 10.1016/j.jbiotec.2008.09.002 18938201

[B19] Slater S Houmiel KL Tran M Mitsky TA Taylor NB Padgette SR Gruys KJ Multiple beta-ketothiolases mediate poly(beta-hydroxyalkanoate) copolymer synthesis in *Ralstonia eutropha* J Bacteriol 1998 180 1979 1987 9555876 10.1128/jb.180.8.1979-1987.1998 PMC107120

[B20] Eschenlauer AC Stoup SK Srienc F Somers DA Production of heteropolymeric polyhydroxyalkanoate in Escherichia coli from a single carbon source Int J Biol Macromol 1996 19 121 130 10.1016/0141-8130(96)01114-2 8842775

[B21] Poirier Y Nawrath C Somerville C Production of polyhydroxyalkanoates, a family of biodegradable plastics and elastomers, in bacteria and plants Biotechnology (N Y) 1995 13 142 150 10.1038/nbt0295-142 9634754

[B22] Aldor IS Kim SW Prather KL Keasling JD Metabolic engineering of a novel propionate-independent pathway for the production of poly(3-hydroxybutyrate-co-3-hydroxyvalerate) in recombinant Salmonella enterica serovar typhimurium Appl Environ Microbiol 2002 68 3848 3854 10.1128/AEM.68.8.3848-3854.2002 12147480 PMC124029

[B23] Madison LL Huisman GW Metabolic engineering of poly(3-hydroxyalkanoates): from DNA to plastic Microbiol Mol Biol Rev 1999 63 21 53 10066830 10.1128/mmbr.63.1.21-53.1999 PMC98956

[B24] Slater S Gallaher T Dennis D Production of poly-(3-hydroxybutyrate-co-3-hydroxyvalerate) in a recombinant *Escherichia coli *strain Appl Environ Microbiol 1992 58 1089 1094 1599234 10.1128/aem.58.4.1089-1094.1992 PMC195559

[B25] Liu SJ Steinbuchel A Exploitation of butyrate kinase and phosphotransbutyrylase from *Clostridium acetobutylicum *for the in vitro biosynthesis of poly(hydroxyalkanoic acid) Appl Microbiol Biotechnol 2000 53 545 552 10.1007/s002530051655 10855714

[B26] Bisswanger H Substrate specificity of the pyruvate dehydrogenase complex from Escherichia coli J Biol Chem 1981 256 815 822 7005225

[B27] Morbach S Sahm H Eggeling L l-Isoleucine Production with Corynebacterium glutamicum: Further Flux Increase and Limitation of Export Appl Environ Microbiol 1996 62 4345 4351 16535457 10.1128/aem.62.12.4345-4351.1996 PMC1388995

[B28] Lee JH Sung BH Kim MS Blattner FR Yoon BH Kim JH Kim SC Metabolic engineering of a reduced-genome strain of Escherichia coli for L-threonine production Microb Cell Fact 2009 8 2 10.1186/1475-2859-8-2 19128451 PMC2634754

[B29] Murarka A Dharmadi Y Yazdani SS Gonzalez R Fermentative utilization of glycerol by Escherichia coli and its implications for the production of fuels and chemicals Appl Environ Microbiol 2008 74 1124 1135 10.1128/AEM.02192-07 18156341 PMC2258577

[B30] Gonzalez-Pajuelo M Andrade JC Vasconcelos I Production of 1,3-propanediol by Clostridium butyricum VPI 3266 using a synthetic medium and raw glycerol J Ind Microbiol Biotechnol 2004 31 442 446 10.1007/s10295-004-0168-z 15378388

[B31] Shams Yazdani S Gonzalez R Engineering Escherichia coli for the efficient conversion of glycerol to ethanol and co-products Metab Eng 2008 10 340 351 10.1016/j.ymben.2008.08.005 18840539

[B32] Lee HC Kim JS Jang W Kim SY Thymidine production by overexpressing NAD+ kinase in an Escherichia coli recombinant strain Biotechnol Lett 2009 31 1929 1936 10.1007/s10529-009-0097-z 19774345

[B33] Debabov VG The threonine story Adv Biochem Eng Biotechnol 2003 79 113 136 12523390 10.1007/3-540-45989-8_4

[B34] Lee KH Park JH Kim TY Kim HU Lee SY Systems metabolic engineering of Escherichia coli for L-threonine production Mol Syst Biol 2007 3 149 10.1038/msb4100196 18059444 PMC2174629

[B35] Conrado RJ Varner JD DeLisa MP Engineering the spatial organization of metabolic enzymes: mimicking nature's synergy Curr Opin Biotechnol 2008 19 492 499 10.1016/j.copbio.2008.07.006 18725290

[B36] Moll JR Ruvinov SB Pastan I Vinson C Designed heterodimerizing leucine zippers with a ranger of pIs and stabilities up to 10(-15) M Protein Sci 2001 10 649 655 10.1110/ps.39401 11344333 PMC2374140

[B37] Dueber JE Wu GC Malmirchegini GR Moon TS Petzold CJ Ullal AV Prather KL Keasling JD Synthetic protein scaffolds provide modular control over metabolic flux Nat Biotechnol 2009 27 753 759 10.1038/nbt.1557 19648908

[B38] Terpe K Overview of bacterial expression systems for heterologous protein production: from molecular and biochemical fundamentals to commercial systems Appl Microbiol Biotechnol 2006 72 211 222 10.1007/s00253-006-0465-8 16791589

[B39] Baba T Ara T Hasegawa M Takai Y Okumura Y Baba M Datsenko KA Tomita M Wanner BL Mori H Construction of Escherichia coli K-12 in-frame, single-gene knockout mutants: the Keio collection Mol Syst Biol 2006 2 2006 0008 10.1038/msb4100050 PMC1681482 16738554

[B40] Datsenko KA Wanner BL One-step inactivation of chromosomal genes in *Escherichia coli *K-12 using PCR products Proc Natl Acad Sci USA 2000 97 6640 6645 10.1073/pnas.120163297 10829079 PMC18686

[B41] Sambrook J Russell D Molecular Cloning: A Laboratory Manual 2001 Third Cold Spring Harbor, NY.: Cold Spring Harbor Laboratory Press

[B42] Tolia NH Joshua-Tor L Strategies for protein coexpression in Escherichia coli Nat Methods 2006 3 55 64 10.1038/nmeth0106-55 16369554

[B43] Abramson J Riistama S Larsson G Jasaitis A Svensson-Ek M Laakkonen L Puustinen A Iwata S Wikstrom M The structure of the ubiquinol oxidase from Escherichia coli and its ubiquinone binding site Nat Struct Biol 2000 7 910 917 10.1038/82824 11017202

[B44] Yeh JI Chinte U Du S Structure of glycerol-3-phosphate dehydrogenase, an essential monotopic membrane enzyme involved in respiration and metabolism Proc Natl Acad Sci USA 2008 105 3280 3285 10.1073/pnas.0712331105 18296637 PMC2265192

